# Review: Mechanotransduction in ovarian cancer: Shearing into the unknown

**DOI:** 10.1063/1.5024386

**Published:** 2018-06-07

**Authors:** Caymen Novak, Eric Horst, Geeta Mehta

**Affiliations:** 1 Department of Biomedical Engineering, University of Michigan, Ann Arbor, Michigan 48109-2800, USA; 2 Department of Materials Science and Engineering, University of Michigan, Ann Arbor, Michigan 48109-2800, USA; 3 Macromolecular Science and Engineering, University of Michigan, Ann Arbor, Michigan 48109-2800, USA

## Abstract

Ovarian cancer remains a deadly diagnosis with an 85% recurrence rate and a 5-year survival rate of only 46%. The poor outlook of this disease has improved little over the past 50 years owing to the lack of early detection, chemoresistance and the complex tumor microenvironment. Within the peritoneal cavity, the presence of ascites stimulates ovarian tumors with shear stresses. The stiff environment found within the tumor extracellular matrix and the peritoneal membrane are also implicated in the metastatic potential and epithelial to mesenchymal transition (EMT) of ovarian cancer. Though these mechanical cues remain highly relevant to the understanding and treatment of ovarian cancers, our current knowledge of their biological processes and their clinical relevance is deeply lacking. Seminal studies on ovarian cancer mechanotransduction have demonstrated close ties between mechanotransduction and ovarian cancer chemoresistance, EMT, enhanced cancer stem cell populations, and metastasis. This review summarizes our current understanding of ovarian cancer mechanotransduction and the gaps in knowledge that exist. Future investigations on ovarian cancer mechanotransduction will greatly improve clinical outcomes via systematic studies that determine shear stress magnitude and its influence on ovarian cancer progression, metastasis, and treatment.

## I. INTRODUCTION

Ovarian cancer is the fifth leading cause of cancer related deaths in females^1^ and remains a deadly diagnosis with 54%^2^ of patients dying from their initial or recurrent diagnosis. While significant advancements in treatment therapies and success rates have been observed in some cancers, there has been no significant progress in ovarian cancer treatment over the past 50 years.^3,4^ Much of this failure arises from the lack of early detection capabilities, with 60%–70% of all patients diagnosed at advanced stages (III or IV),^1,5–8^ and an 85% recurrence rate.^9^ Ovarian cancer is categorized by the cell of origin, with approximately 90% originating from epithelial cells. Epithelial ovarian cancers arise from either an ovarian surface epithelial stem cell or a fimbrial stem cell that becomes entrapped within the ovary cortex. This entrapped cell then forms a cortical inclusion cyst that is driven to high-grade serous carcinoma from the aberrant niche environment.^3,10,11^ The readers are requested to refer to the review by Ng and Barker for the detailed origin of ovarian cancers.^10^ Epithelial ovarian cancer is classified into histological subgroups, where serous carcinoma makes up 70% of all tumors.^11^ The serous histological subtype is grouped into a two-tier system based on the prevalence of mitotic rate and atypical nuclei.^3,12^ 90% of all serous epithelial ovarian cancer is of “high grade,” making it the most prevalent type of ovarian cancer characterized by TP53 mutations, rapid tumor growth, and high recurrence.^11,12^ The recurrent disease is often chemoresistant and has a median survival of 12–24 months.^9^ Detection of ascites within the peritoneal cavity is associated with most stages of ovarian cancer. According to the American Joint Committee on Cancer (AJCC) and International Federation of Gynecology and Obstetrics (FIGO), stage IC, IIB, III, and IV ovarian cancers are all categorized by the presence of cancer in the peritoneal cavity.^13,14^ The detection of malignant ascites is an integral step in the clinical assessment of ovarian cancer.^15^ Furthermore, malignant ascitic fluid is a major contributor to ovarian cancer progression and poor prognoses,^16^ and is consequently closely monitored by oncologists. Many of these statistics arise from factors within the tumor microenvironment; therefore, it is critical to consider their role when striving to understand and devise treatment strategies to improve patient outcomes. This review will address the contribution of specific cues from the tumor microenvironment to the disease progression and the impact of these findings on our understanding of ovarian cancers.

### A. The ovarian cancer mechanical microenvironment

Located within the peritoneal cavity, the ovaries exist within the abdominal space where the cellular and acellular content are tightly regulated by the anatomy of the peritoneal membrane. The peritoneal membrane consists of five layers: endothelial cells, endothelial basement membrane, interstitial space, submesothelial basement membrane, and mesothelial cells.^17^ These tight layers inhibit cells and large protein molecules such as albumin from migrating into the peritoneal cavity. In healthy individuals, the peritoneal membrane modulates a net oncotic pressure out of the cavity^17^ filtering 50–100 ml of fluid into the lymphatic vessels every hour,^18^ with post-menopausal women carrying an average of 2.3 ml of intraperitoneal fluid at any given time.^19^ However, in a diseased state, this intraperitoneal fluid is not readily drained and a backup of liquid, termed ascites, may begin to amass in some patients.

Approximately, 36.7% of all ovarian cancer patients develop ascites,^20–22^ defined as a minimum of 25 ml of fluid accumulation^23^ within the peritoneal cavity. The retention of ascitic fluid in diseased patients is predicted to stem from an increase in the permeability of the capillaries through the peritoneal membrane, lymphatic obstruction of normal drainage, and the net oncotic pressure into the cavity.^16–18,24^ Ovarian cancer cells and cellular aggregates that are shed into the peritoneal cavity can physically block the homeostatic lymphatic drainage system.^25^ This theory of ascitic fluid retention in ovarian cancers has been around for more than 60 years;^24,26,27^ yet, the exact mechanisms have yet to be proven.^16^

**FIG. 1.**
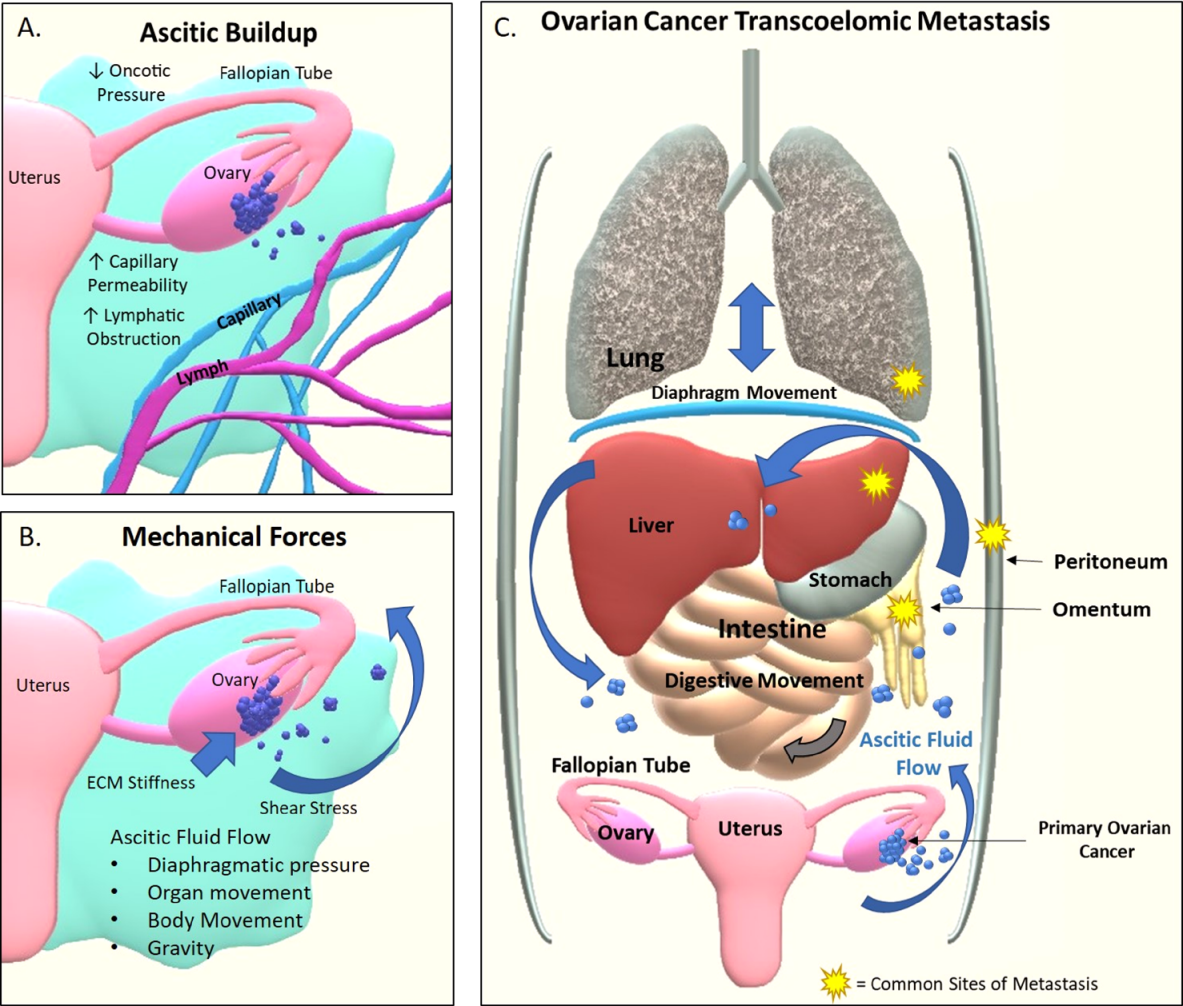
The microenvironment of ovarian cancer facilitates transcoelomic metastasis. (a) The buildup of ascites is triggered by the primary tumor which causes increased capillary permeability, lymphatic obstruction of drainage, and an overall decrease in oncotic pressure out of the peritoneal cavity. (b) The ovarian cancer cells experience the surrounding ECM stiffness within the primary tumor, spheroid cell aggregates within the ascites, and potential metastatic sites. Shear stress stimulates the ovarian cancer cells via interstitial fluid flow within the primary tumor and ascitic fluid flow triggered by gravity, bodily movements, change in the diaphragmatic pressure from breathing, and organ movements from functions such as digestion. (c) Transcoelomic metastasis starts with the exfoliation and detachment of cancer cells from the primary tumor site caused by shear stress within the ascites. Cancer cells within ascites evade the immune system and detached cells form spheroids to avoid anoikis. Ovarian cancer spheroids are then carried by the ascitic current to metastatic sites where implantation, invasion, and growth facilitate the formation of new tumors.

The presence of ascitic fluid has been shown to aid in metastasis^28^ and chemoresistance.^29,30^ It also mechanically stimulates the cancer with hydrostatic compression and shear forces. The ascitic fluid flow is triggered by gravity, changes in diaphragmatic pressure from breathing, surrounding organ movement aiding digestion, and bodily movements like walking.^31^ The continuous barrage of turbulent fluid flow stimulates a variety of mechanotransduction signaling pathways and further exfoliates ovarian tumor cells and cellular aggregates from the ovarian surface epithelium into the peritoneal cavity. After their escape into the ascites, these free-floating cancer cells and cellular clusters often self-assemble and aggregate to form spheroids, thereby overcoming anoikis.^25,32^ Once ovarian cancer cells have disseminated within the ascites, they have access to the most common metastatic sites of ovarian cancers: the peritoneum, the greater omentum, the right subphrenic region, the lung, and the liver.^31–33^ The presence of ascites and forces associated with them facilitate transcoelomic metastasis, the most common form of ovarian cancer metastasis.^28,31^ Figure 1 details the mechanical forces relevant to ovarian cancers, the ascitic buildup, and the transcoelomic metastatic process in ovarian cancers.

Ovarian cancer cells isolated from ascites are rich in cancer stem cells (CSCs).^34,35^ CSCs are defined as a small subset of cancer cells, with the capability of self-renewal, multilineage differentiation, tumor initiation, metastasis, and chemoresistance to conventional or targeted chemotherapies and radiotherapies. Ovarian CSCs are typically identified through expression of specific markers such as CD133, ALDH1A, CD24, CD117, CD44,^36–39^ and micro ribonucleic acid (miRNA), as well as functional phenotypes such as self-renewal, production of heterogeneous progenies, and enhanced tumor formation capabilities.^36^ CSCs are typically enriched after chemotherapy as residual cells that lead to tumor relapse in patients. The presence of ascites increases the drug efflux mechanisms within the ovarian cancer cells including ABC transporter genes: *MDR1a*, *MDR1b*, and *BCRP.*^34,40^ The upregulation of these transporter genes provides ovarian cancer cells the necessary mechanisms to survive chemotherapy and renew tumor growth post-treatment. Additionally, ascites have been shown to enhance epithelial to mesenchymal transition (EMT) in ovarian cancer cells.^8,41,42^ During EMT, a stationary epithelial cell transforms into a mesenchymal cell capable of motility. This transition is an important precursor for metastasis and chemoresistance.^43,44^ Currently, the role of mechanical cues within the ovarian tumor microenvironment that leads to these outcomes is not well defined. Therefore, the effects of mechanotransduction in the ovarian cancer microenvironment need to be investigated in the context of disease progression and chemoresistance. It is likely that future findings could greatly improve patient treatment and outcome. The known contribution of mechanical cues towards tumor progression and metastasis within the ovarian cancer microenvironment is reviewed in Secs. II B, II D, and III.

## II. 2D AND 3D *IN VITRO* MODELS OF OVARIAN CANCER MECHANOTRANSDUCTION

To study the physiologically relevant forces of shear stress and extracellular matrix (ECM) stiffness, many research groups have developed bioreactors capable of systematic and controlled force stimulation that independently explore the effects of mechanical stimuli on ovarian cancer. Here, we detail the published studies that have investigated the effects of mechanical stimuli on ovarian cancer and their overall findings.

### A. Shear stress estimates in ovarian cancers

Accurate *in vivo* shear force estimates within patient ascites and the corresponding shear stress values on ovarian cancer are not known. It has been predicted that shear stress values within ascites are low, with relatively little to no support in either experimental or mathematical modeling.^29,31,45^ Computer simulated models are required to improve our understanding of the physiological stresses that occur within the peritoneal cavity. A diseased patient's musculoskeletal/organ movements cause a change in the shape of the peritoneal cavity which in turn causes fluid movement within the ascites. This fluid movement is directly correlated to the levels of shear stress experienced by both free floating and attached ovarian cancer spheroids. These complex multistep interactions can be modeled with the help of finite element analysis and fluid dynamic modeling systems. The interstitial fluid velocity ranging from 0.2 to 0.8 *μ*m/s has been reported in neoplastic tissues,^46^ but no direct measurements of ovarian specific tissues exist. Moreover, the wall shear stress in a computational simulation of gastrointestinal models^45^ ranges from 0.14 to 11 dyn/cm^2^,^45,47^ and has been used as an estimate for shear stress ranges on ovarian tumors. In contrast, circulating tumor cells experience a large range of shear stresses from venous (0.5–4.0 dyn/cm^2^) and arterial blood flow (4.0–30.0 dyn/cm^2^).^48^ Given the paucity of research on the physiological role of shear stress and specific values relevant to ovarian cancer, there is a critical unmet need for systematic studies that determine shear stress magnitude and its influence on ovarian cancer progression, metastasis, and treatment.

### B. Shear stress models specific to ovarian cancer

**FIG. 2.**
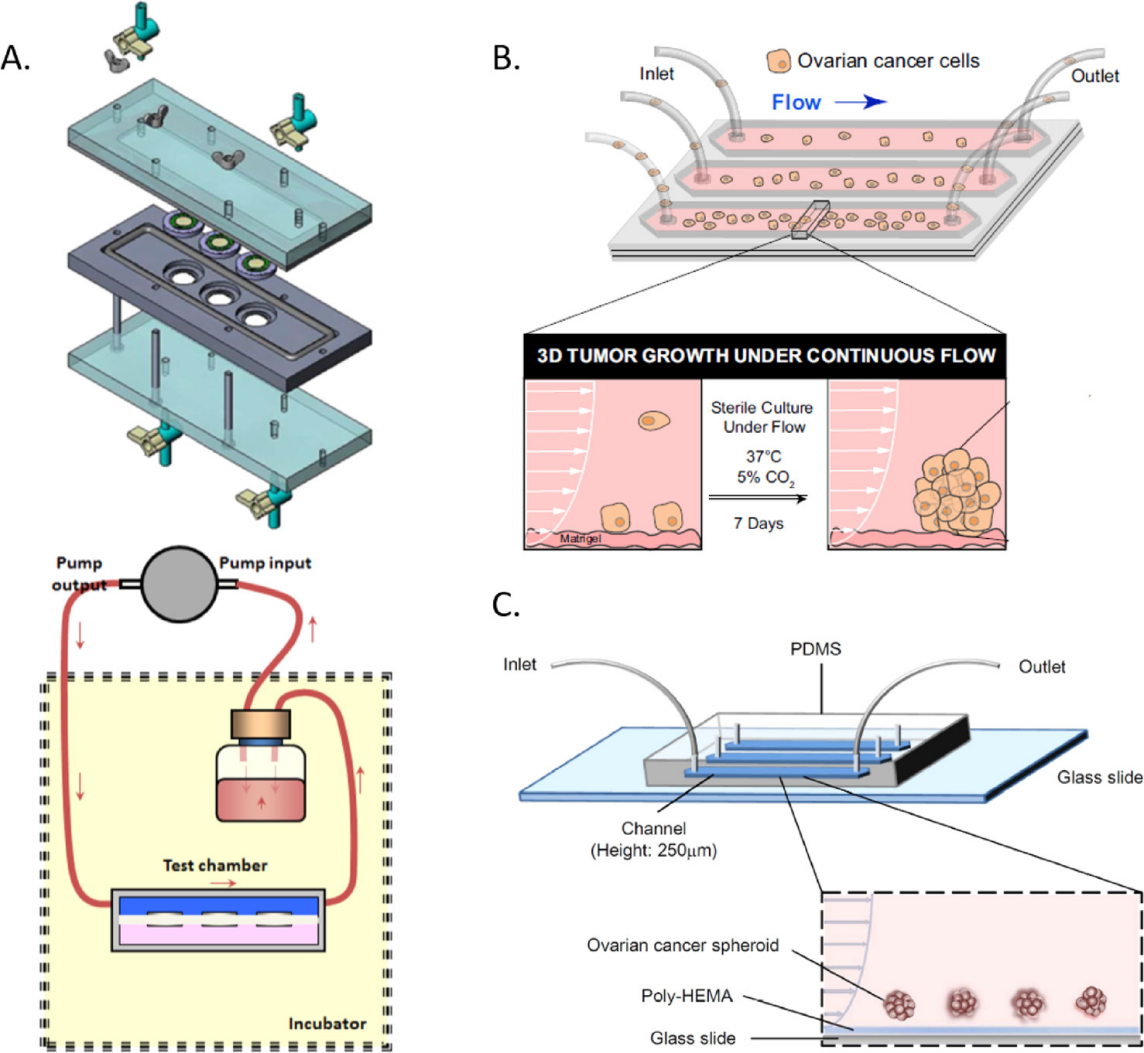
Selected bioreactors and devices utilized for ovarian cancer shear stress investigations. (a) Flow chamber schematic of the 2D ovarian cancer cell culture using a closed circuit pump design for shear stress stimulation.^45^ (b) 2D/3D hybrid design where ovarian cancer cells flow into the microfluidic chamber, adhere to the Matrigel basement layer, and continue to grow under a shear stress stimulus for 7 days.^31^ (c) Microfluidic device design where ovarian cancer spheroids do not adhere to the poly-HEMA basement layer and are stimulated with shear stress within the channel for 24 h.^29^ Reproduced with permission from Ip *et al.*, Sci. Rep. **6**, (2016). Copyright 2016 Nature Publishing Group,^29^ Avraham-Chakim *et al.*, PLoS One **8**, e60965 (2013). Copyright 2013 PLOS,^45^ and Rizvi *et al.*, Proc. Natl. Acad. Sci. **110**, E1974–E1983 (2013). Copyright 2013 National Academy of Sciences.^31^

The study of shear stress on cells has been considerably investigated with both commercially available and custom-made lab bioreactors.^29,49–51^ However, only a few published studies have investigated shear stress stimulation of ovarian cancer cells in 2D or 3D culture models. To answer the question of how fluid flow induced wall shear stress affects the cytoskeleton of ovarian cancer and regulates its penetration and spread to the peritoneum, Avraham-Chakim *et al.* fabricated a custom-made 2D shear stress device^45^ [shown in Fig. 2(a)]. OVCAR3 cells, representative of high grade serous ovarian cancer,^52^ were cultured in monolayers and then exposed to shear stress of 0.5–1.5 dyn/cm^2^ for 30 min. Morphological analysis revealed that the shear stimulated OVCAR3 cells elongated significantly, increased stress fiber formation, and generated a cytoskeletal network of microtubules with increasing shear stress. Shear stress experienced by ovarian cancer cells induced cell motility and targeting these specific cytoskeletal pathways may benefit ovarian cancer treatment.^45^

Seeking to replicate the initial dissemination of ovarian cancer cells into the peritoneal cavity, Hyler *et al.* devised an experiment to test low levels of shear stress on five cell lines of variable metastatic potential. Three murine ovarian cell lines ranging from benign to highly aggressive mouse ovarian cancer epithelial cells (MOSE), OCE1 (benign human), and SKOV3 (human ovarian clear cell adenocarcinoma) cell lines were exposed to fluid shear stress ranging from 0.13 to 0.32 dyn/cm^2^ on a rotator plate for up to 12 days.^53^ Fluid shear stress was shown to increase the capacity for spheroid formation in cell lines with a higher metastatic phenotype, increase the number of actin-containing protrusions and vinculin-containing focal adhesions for all cell types, as well as show nuclear change with an increase in multi-lobed nuclei and the number of tetraploid chromosomes in benign cell populations.

Molecular changes associated with the metastatic cascade due to continuous shear force was investigated within a microfluidic device designed by Rizvi *et al.*^31^ [Fig. 2(b)]. High grade serous ovarian cancer OVCAR5 cells in suspension were placed under continuous flow for 7 days above a Matrigel basement layer used to model a stromal bed. The shear stress varied with the location within the device, with the flow velocity ranging from 0 mm/s on the edge of the device to approximately 10 mm/s throughout the device center, where majority of cell attachment was located. The cells that attached under these shear conditions formed micronodules and showed increased EMT biomarkers, including decreased proliferation, upregulation of epidermal growth factor receptor (EGFR), decreased E-cadherin expression, and an associated increase in vimentin expression without any change in integrin α5.^31^ The flow-induced EMT was predicted to influence the chemoresistance of cells and the effectiveness of targeted inhibitors. These predictions were sequentially validated by Ip *et al.*^29^

Expanding upon the previous findings of Rizvi *et al.*, Ip *et al.* sought to identify the role of CSC in ovarian cancer chemoresistance. SKOV3, a p53 mutant clear cell adenocarcinoma cell line, was first grown into spheroids before being placed under extremely low shear conditions (0.02 and 0.002 dyn/cm^2^) in a microfluidic shear device^29^ [Fig. 2(c)]. Shear stress was applied to the spheroids atop a poly(2-hydroxyethyl methacrylate) (Poly-HEMA) layer, to prevent adherence, for 24 hours, before sequential analysis was performed. The lack of adherence to the basement layer provided 3D stimulation mimicking that of the ascitic environment. The shear stimulated SKOV3 spheroids were found to have enriched with CSCs with the expression of Oct-4, CD117, ABCG2, and P-gp. Concurrently, EMT was enhanced through the upregulation of gene and protein expression of Snail, Slug, and N-cadherin, and downregulation of E-cadherin.^29^ Apart from substantiating the work of Rizvi *et al.*, they found that the shear stress stimulated cells were chemoresistant to cisplatin and paclitaxel treatment, as previously hypothesized.^31^ The CSC phenotypes and chemoresistance were attributed to the PI3K/Akt signaling pathway, where LY294002, a specific inhibitor to PI3K, abated the previously observed enhanced CSC marker expression. Sequential chemotherapy treatment was not performed with the PI3K/Akt inhibitor. Overall, these findings emphasize the impact that shear stress stimulus has on chemoresistance and recurrence through CSC populations within patient ascites. These findings also bring to light the importance of the PI3K/Akt pathway suggesting it as an essential target within CSC and chemoresistant phenotypes in ovarian cancer, though additional work should be done to validate these findings in additional cell lines.

The spread of ovarian cancer to distant metastatic sites through tumor cells that have intravasated to the circulation and then extravasate and colonize a new tumor site was investigated by Egan *et al.*^54^ and Giavazzi *et al.*^55^ Egan *et al.* utilized a simple cone and plate viscometer setup to test the protection potential of platelets when sheared under venous and arterial stresses with A2780 (endometrioid histotype) ovarian cancer cells. This setup was designed to test the viability of circulating tumor cells under physiological shear stresses within arterial and venous circulation. This is an important point of concern once tumor cell extravasation has occurred; however, it is a less predominant form of ovarian cancer metastasis. Shear rates of 1.5 and 12 dyn/cm^2^ were explored for 10 minutes with and without platelet incorporation. The amount of lactate dehydrogenase (LDH) was measured for the indication of cancer cell membrane damage. The results demonstrated a significant reduction in LDH when platelets were present under shear stress, implying the prolonged non-destructive circulation of cancer cells under *in vivo* conditions.^54^

Beyond circulation survival, the ability to adhere and extravasate is necessary for circulating tumor cells to metastasize. The rolling and attachment capability of circulating tumor cells was investigated by Giavazzi *et al.* where a 2D/3D hybrid approach was developed from a parallel plate apparatus. This experimental design was developed to determine ovarian cancer cell affinity to adherence and rolling on a 2D culture of human umbilical vein endothelial cells (HUVEC). This design contained OVCAR3 cells within a fluidic suspension and the shear stress ranged from 0.3 to 3.0 dyn/cm^2^ to more closely replicate venous blood flow for a duration of 12 min. Only a small proportion of their experiments pertained to OVCAR3 cells, but results showed that little interaction occurred between the resting HUVEC surface layer and OVCAR3 cells, while minimal attachment and rolling occurred on IL-1 activated HUVECs.^55^ These findings implicate that specific adhesion mechanisms are necessary for ovarian tumor cell attachment and extravasation, while the cell type also plays a critical role in which attachment or rolling mechanisms are utilized. A compact summary of the shear stress mechanotransduction studies on ovarian cancer is detailed in Table I with schematics of select bioreactors shown in Fig. 2.

**TABLE I.** Ovarian cancer specific shear stress investigations and major findings.

2D/3D culture	Device design	Shear stress and duration	Cell type	Findings	Citation
2D/3D hybrid	Parallel plate	0.3–3.0 dyn/cm^2^ 12 min	OVCAR3	• Little interaction with HUVEC resting cells • Some attachment and rolling on IL-1 activated HUVEC cells	Giavazzi *et al.*^55^
			HUVEC monolayer		
2D	Rotator plate	0.13–0.32 dyn/cm^2^ 12 days	MOSE-E	• Increased spheroid formation• Formation of actin-containing protrusions• Increase in vinculin-containing focal adhesions• Change in nuclear structure associated with aneuploidy	Hyler *et al.*^53^
			MOSE-L		
			MOSE-L_TICν_		
			OCE1		
			SKOV3		
2D	Custom	0.5, 1.0, 1.5 dyn/cm^2^ 30 min	OVCAR3	• Cell elongation• Formation of stress fibers• Formation of cytoskeletal microtubule network	Avraham-Chakim *et al.*^45^
2D/3D hybrid	Custom microfluidic	Range of 0 to >10 mm/s 7 days	OVCAR5	• Increased EMT • Increased EGFR, vimentin, p27Kip1 • Decreased E-cadherin, CDC2	Rizvi *et al.*^31^
3D	Cone and plate viscometer	1.5, 12 dyn/cm^2^ 10 min	A2780	• Reduced lactate dehydrogenase (LDH) release with platelet co-culture under shear	Egan *et al.*^54^
3D	Custom microfluidic	0.02, 0.002 dyn/cm^2^ 24 h	SKOV3	• Enhancement of CSC markers: Oct-4, CD117, ABCG2, P-gp• Increased EMT• Enhanced chemoresistance• PI3K/Akt signaling pathway involvement	Ip *et al.*^29^

### C. Alternative shear bioreactors for examining ovarian cancer mechanotransduction

Cancer induced ascites or malignant ascites are not unique to ovarian cancer. Other cancers, including colon, pancreatic, gastrointestinal tract, lung, and breast, feature tumor cells in ascites and pleural effusion.^17,56^ Tumor cells within the ascites are often found at late stages of cancer progression. Previous studies have investigated the impact of shear stress stimulus on a variety of cancer types due to ascitic shear stresses, heightened interstitial fluid flow, and high shear conditions experienced by circulating tumor cells.^57^ For a review of shear stress studies on cancer, the readers are kindly referred to Mitchell and King.^57^

Work on breast cancer has shown shear stress to affect: adherence to the endothelium^58^ due to an increase in the expression of EMT characteristics,^51^ acidic microenvironment development,^59^ cancer stem cell populations,^60^ migration,^61,62^ involvement of caveolin-1 through the FAK/Src, ROCK/pMLC,^52^ and PI3K/Akt/mTOR^63^ pathways, and glycoprotein IIb/IIIa and αvβ3 integrin in PI3K/Akt and NF-kB signaling.^64^ Glioma cells exposed to shear stress showed migratory activity dependent on matrix metalloproteinase (MMP) activation and expression,^65^ while prostate cancer cells showed YAP1 dependent motility.^66^ Shear stress stimuli on bladder, colon, and pancreatic cancers have shown enhanced axial spreading,^67^ sensitization to TRAIL-induced apoptosis,^49^ involvement of Wnt/β-catenin, mitogen-activated protein kinase (MAPK), and NF-κB pathways,^68^ and the necessity of mucin 16 for pancreatic cell adherence.^69^

**FIG. 3.**
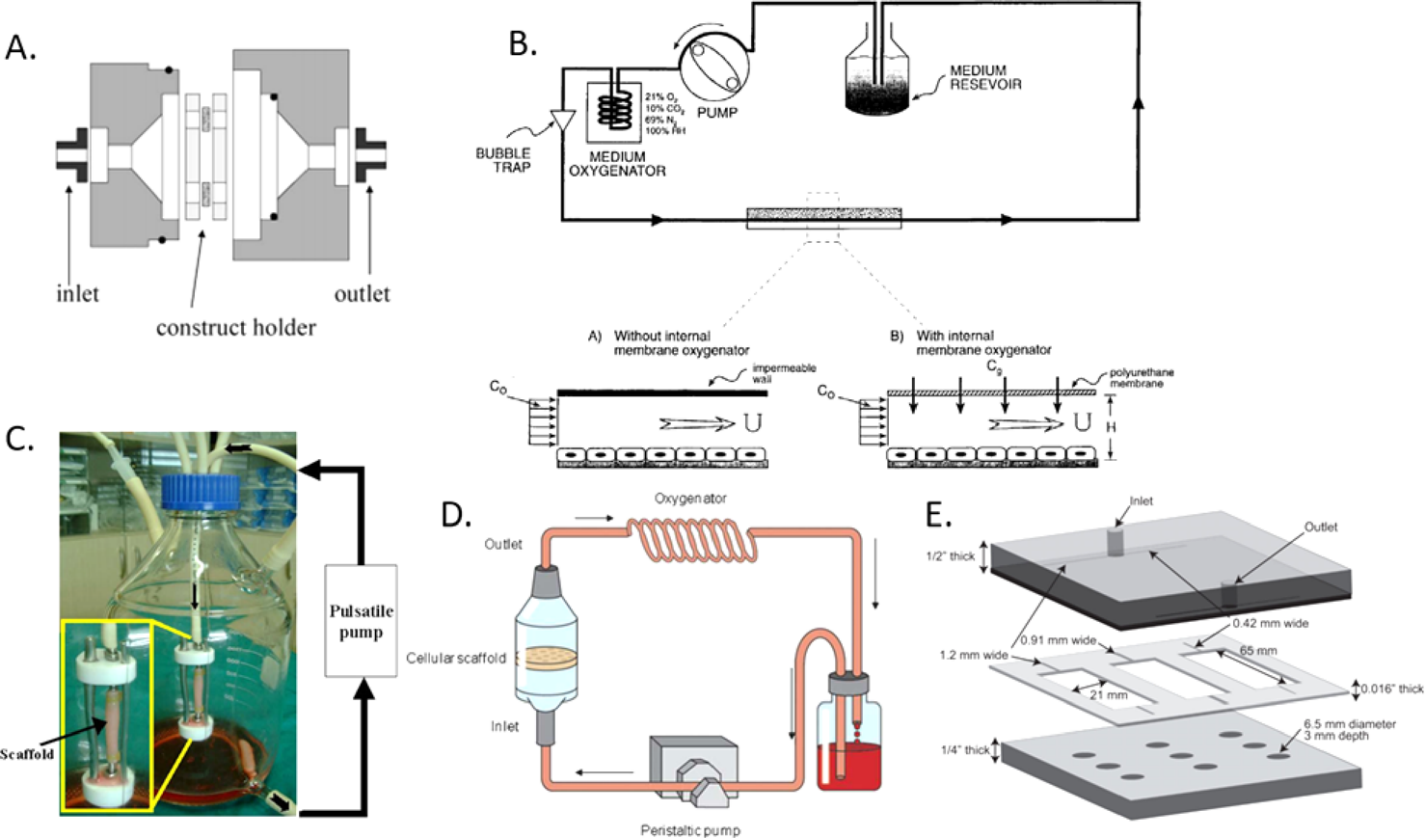
Relevant shear stress bioreactors for future studies on ovarian cancer mechanotransduction. (a) Custom 3D porous scaffold shear bioreactor device; cells were seeded on a 1 mm thick biodegradable polyester-urethane foam and perfused with medium.^70^ (b) 2D flat plate design; cells were seeded on a glass slide and experienced uniform fluid shear.^71^ (c) 3D shear bioreactor utilizing a PLCL tubular scaffold; cells were seeded onto a particulate leached PLCL scaffold and perfused with medium.^72^ (d) Porous perfusion scaffold bioreactor; cells were seeded onto a perfused particle leached PLLA/PLGA scaffold.^73^ (e) 3D microfluidic device providing three unique shear rates; cells seeded on a Collagen Type I scaffold experienced shear over the surface of the scaffold.^74^ Reproduced with permission from Raimondi *et al.*, Biorheology **43**, 215–222 (2006). Copyright 2006 IOS Press,^70^ Tilles *et al.*, Biotechnol. Bioeng. **73**, 379–389 (2001). Copyright 2001 John Wiley & Sons,^71^ Lesman *et al.*, Biotechnol. Bioeng. **105**, 645–654 (2010). Copyright 2010 John Wiley & Sons,^73^ Jeong *et al.*, Biomaterials **26**, 1405–1411 (2005). Copyright 2005 Elsevier,^72^ and Mahler *et al.*, Biotechnol. Bioeng. **111**, 2326–2337 (2014). Copyright 2014 Elsevier.^74^

Given that the ascitic environment has been investigated for other tumor cell types, these findings may be of interest to future investigations in ovarian cancer mechanotransduction. The specific pathway findings such as involvement with PI3K, Akt, ROCK, and NF-κB must be considered, as PI3K/Akt pathway contributions under shear stress have already been identified.^29^ Additionally, CSC populations, migration potential, and metastatic potential should all be scrutinized under shear stress because of the concurrent findings between cell types. However, novel studies on ovarian cancer cells, including those derived from primary and metastatic tumors and ascites, are still needed to confirm these similarities and identify the unique characteristics and potential target pathways for ovarian cancer mechanotransduction. Distinct bioreactor designs will arise depending on specific biological questions. Shear bioreactors have been implemented in cell culture for the past quarter-century. Their designs have ranged from 2D microfluidic devices to large scale 3D perfusion bioreactors. With these devices, researchers have been able to test shear forces on cells seeded on a wide variety of surfaces and scaffolds. However, each shear stress bioreactor device also comes with a specific set of design limitations that must be taken into consideration when devising an experiment. For example, some devices have a limited working shear stress range. In the case of ovarian cancer, it is currently hypothesized that most shear stresses experienced in the peritoneal cavity are below 1 dyn/cm^2^.^29^ Some bioreactors may not be suitable for providing this type of shear stress value, especially in a manner that is both consistent and reproducible. Bioreactors such as the orbital shaker and the cone and plate viscometer will have intrinsic variable shear stress and may produce shear ranges outside that of suitable physiological values. Other bioreactors may only support 2D culture, making it impossible to incorporate any type of 3D scaffold within them. Table II details some popular shear bioreactor designs and schematics of select bioreactors are shown in Fig. 3. The application of these devices to ovarian cancer investigations may be suitable for future research.

**TABLE II.** Prominent shear stress bioreactors: Shear stress bioreactors with design relevance for future investigations in ovarian cancer research.

2D/3D culture	Material and device	Stimulant type	Shear stress	Cell type	Citation
2D	Flat plate	Laminar flow	0.01–21 dyn/cm^2^	Rat hepatocytes cocultured with 3T3-J2 fibroblasts	Tilles *et al.*^71^
2D	Cone and plate	Laminar flow	5 dyn/cm^2^	Human endothelial cells	Dai *et al.*^75^
2D	Orbital shaker	Laminar flow	5–14 dyn/cm^2^	Endothelial cells	Dardik *et al.*^76^
2D	Tubular poly(ethylene glycol) (PEG) microfluidic device	Laminar flow	0.5 dyn/cm^2^	PC3 prostate cancer cells	Lee *et al.*^66^
3D	Poly(lactide-*co*-caprolactone) (PLCL) tubular perfusion bioreactor	Laminar porous flow	Flow rate: 130 ml/min, P = 25 mmHg 1 Hz pulse	Rabbit aortic smooth muscle cells	Jeong *et al.*^72^
3D	Polyester-urethane foam perfusion bioreactor	Laminar porous flow	0.046–0.56 dyn/cm^2^	Bovine articular chondrocytes	Raimondi *et al.*^70^
3D	Porous poly(l-lactic acid)/poly(l-lactic-*co*-glycolic acid) (PLLA/PLGA) scaffold perfusion bioreactor	Laminar porous flow	1–10 dyn/cm^2^	Human foreskin fibroblasts	Lesman *et al.*^73^
3D	Alginate scaffold perfusion bioreactor	Laminar porous flow	1–13 dyn/cm^2^	Human umbilical vein endothelial cells	Rotenberg *et al.*^49^
3D	Collagen type I gel microfluidic device	Laminar flow or oscillatory shear	2–20 dyn/cm^2^	Porcine aortic valve endothelial cells	Mahler *et al.*^74^

### D. ECM stiffness within the ovarian cancer mechanical microenvironment

An additional prominent feature of a cell's mechanical microenvironment is the rigidity of its ECM. Cells can perceive the surrounding stiffness of their microenvironment and its modulation has been shown to heavily influence phenotype,^77,78^ protein expression,^79,80^ and differentiation.^81,82^ For cancer cells, the stiffness of their surrounding ECM can influence metastasis, invasion, proliferation, and chemoresistance.^83–86^ Numerous studies have proven that stiffer substrates enhance the metastatic phenotypes of cancer cells.^87–91^ However, within the field of ovarian cancer, studies have resulted in contradictory findings.

To examine the impact of compliant versus rigid ECM stiffness, McGrail *et al.* first differentiated human mesenchymal stem cells (MSCs) into either adipocytes or osteoblasts via substrate stiffness. The resulting cell monolayers had differential innate stiffness values, E = 0.9 kPa or E = 2.6 kPa, respectively. These cell layers were then used to analyze ovarian cancer cell preference for adherence and migration patterns. Ovarian cancer cells were found to be more adherent to softer adipocyte substrates with enhanced migratory capacity, as well as being more proliferative and chemoresistant, despite predictions. The Rho-ROCK signaling pathway was crucial to these phenotypic observations. EMT traits were observed on the soft adipocyte cultures where SKOV3 cells exerted traction force and showed an elongated morphology indicating a mesenchymal phenotype. When results were compared to the less metastatic cell line OVCAR3, enhanced adhesion, proliferation, chemoresistance, and migration on the soft substrates were not observed. The OVCAR3 cells only displayed a slight increase in traction forces. Treatment with lysophosphatidic acid (LPA), an activator of Rho and ROCK, induced motility on stiff substrates and collapse of the cells on soft substrates due to hypercontractility. The specific inhibition of ROCK by small-molecule inhibitors Y27632 and H1152 lead to rigidity independent mobility of the ovarian cancer cells. These findings demonstrated the importance of substrate stiffness on ovarian cancer cell phenotype, differing metastatic potentials between cell lines, and the incorporation of the Rho/ROCK pathway in ovarian cancer mechanotransduction.^92^

To evaluate the importance of investigating cellular-ECM interactions in a 3D environment, varying stiffness 3D constructs were studied by Zhang *et al.*,^93^ Loessner *et al.*,^94^ and Guo *et al.*^95^ The work of Zhang and Loessner both utilized PEG constructs. Zhang *et al.* investigated hydrogels with three stiffnesses and found that the epithelial ovarian papillary serous cyst-adenocarcinoma cell line,^96^ HO8910, grew the fastest, formed multicellular spheroids, and adhered preferentially to the medium hydrogel stiffness, of 12 kPa.^93^ The PEG gel investigated by Loessner *et al.* incorporated both MMP cleavable sites and arginylglycylaspartic acid (RGD) motifs to enhance cell attachment and allow cell motility. The 3D cultures formed spheroids and exhibited higher chemoresistance in 3D vs 2D culture. Enhanced proliferation was found in the 2D cultures and OV-MZ-6 3D cultures (a serous adenocarcinoma ovarian cancer cell line).^97^ Within 3D culture, cells increased the expression of α3/α5/α1 integrin surface receptors as well as MMP9 production. Greater proliferation was found on RGD or MMP functionalized hydrogels compared to the PEG gels alone, and less proliferation was found on stiffer hydrogel constructs.^94^ The contradictory finding of enhanced proliferation and cell aggregation within the stiffer constructs was observed in an investigation by Guo *et al.*^95^ As the 3D culture material used in this study consisted of crosslinked egg whites as opposed to PEG hydrogels, the conclusions from this study are not directly comparable^94^ to those of Zhang and Loessner *et al.*

Overall, these findings point towards a preference of softer substrates for ovarian cancer growth and metastatic advancement. A detailed layout of the experiments and conclusions for ovarian cancer stiffness effects can be found in Table III. Given the minimal number of ovarian cancer ECM stiffness investigations and contradictory evidence, further studies are needed to deepen our understanding of the role of substrate stiffness in ovarian cancer mechanotransduction.

**TABLE III.** Ovarian cancer specific stiffness investigations and major findings.

2D/3D culture	Material	Stiffness (kPa)	Cell type	Findings	References
3D	PEG hydrogel with RGD and MMP degradable motifs	12.01, 0.241	OV-MZ-6 SKOV3	• 3D culture • Spheroid formation • Higher chemoresistance • Increased expression: a3/a5/b1 integrins and MMP9 • Less proliferation in stiffer gels • Greater proliferation in RGD or MMP functionalized hydrogels • 2D culture • Enhanced proliferation	Loessner *et al.*^94^
3D	PEG crosslinked poly(vinyl ether-*co*-maleic acid) hydrogel	2.19–105.1	HO8910	• Multicellular spheroid formation• Gel with 12.02 kPa stiffness • Fastest cell growth • Best cell adherence	Zhang *et al.*^93^
2D	Human mesenchymal stem cells differentiated to soft and stiff adipocytes and osteoblast monolayers on polyacrylamide substrates	Adipocytes (E = 0.9)	SKOV3 OVCAR3	• SKOV3 on soft substrate • Increased adherence to softer substrates • More proliferative and chemoresistant • Enhanced EMT and traction forces • Elongated morphology• OVCAR3 on soft substrate • Slight increase in traction forces• Rho/ROCK dependent phenotypes	McGrail *et al.*^92^
		Osteoblasts (E = 2.6)			
		Polyacrylamide: 2.83, 34.88			
3D	Egg white and poly[(methyl vinyl ether)-alt-(maleic acid)]	G′ range	SKOV3	• Enhanced proliferation in stiffer samples• Greater cell aggregation in stiffer samples	Guo *et al.*^95^
		0.00121–0.06328			
		G″ range			
		0.00043–0.01362			

It may be beneficial to consider the prominent pathways affected by ECM stiffness in other cancer malignancies as potential starting points of investigation in ovarian cancers. Some prominent pathways modulated by substrate stiffness in cancer include YAP/TAZ, Rho/ROCK, Cav1, and FAK/PI3K/Akt. The transcription factors YAP (Yes-associated protein) and TAZ (transcriptional coactivator with a PDZ-binding motif) have been shown to be heavily associated with ECM stiffness, cell spreading, and stress fiber activity.^98–100^ Additionally, YAP/TAZ is implicated in many important cancer hallmarks including proliferation, metastasis, and stem cell-like behavior.^101,102^ As ovarian cancer experiences an environment with variable stiffness, the YAP/TAZ pathway is a point of interest for future mechanotransduction studies. The Rho/Rock pathway has already been tied to stiffness effects on ovarian cancer cells^92^ and it has been established as a well-known factor in both mechanotransduction and cancer progression for a variety of tumor types.^103–107^ Caveolin-1 has been shown to be essential for stiffness sensing, and thus when silenced, tumor cells are able to proliferate and migrate independent of the rigidity of the surrounding ECM.^108^ However, these claims appear dependent on the cancer cell type, as confounding evidence has been demonstrated regarding their contribution to tumor growth and metastasis.^109–111^ Ovarian cancer studies concerning Cav-1 have shown it to be downregulated in both primary cells and immortalized cell lines, indicating its likely action as a tumor suppressor.^112–115^ However, these studies have yet to correlate Cav-1 to ECM stiffness. Upregulation of the FAK-PI3K/Akt pathway has been attributed to enhanced ovarian cancer migration and invasion.^116^ It is also a known pathway in mechanotransduction activation through stiffness modulation.^117^ Therefore, future ovarian cancer studies must study the activation of this pathway in conjunction with ECM stiffness.

Most mechanotransduction pathways involving stiffness are highly integrated, thereby making them quite complex, and as a result, difficult to study. However, the correlation that ECM stiffness has with cancer metastasis also makes it a promising avenue for new and innovative ovarian cancer treatments. As a complete examination of cancer mechanotransduction pathways is beyond the scope of this review, additional details on the influence of ECM stiffness can be found in the works by Pathak and Kumar,^87^ Spill *et al.*,^101^ and Chin *et al.*^118^

## III. RELATING *IN VITRO* MECHANOTRANSDUCTION RESULTS TO *IN VIVO* PATIENT OUTCOMES

The exploration of mechanotransduction within ovarian cancer is still in its infancy. However, current findings reiterate the urgency of expanding this field for furthering the development of drug targets within metastasis, chemoresistance, and tumor recurrence pathways. The overlap of clinical and laboratory based findings consistently hint at the important role of mechanotransduction in the progression of ovarian cancer.

The direct impact of mechanotransduction on ovarian cancer and its associated pathways remains vastly unknown both *in vitro* and *in vivo*. Clinical research has shown that side populations of ovarian cancer cells found within the ascites can display the characteristics of both EMT and stem cell-like behavior.^119–121^ EMT is an important part of ovarian cancer progression, in which free floating spheroids attach to the mesothelium, disseminate and metastasize to surrounding tissues.^122,123^ Expression of CD44 and CA125, high levels of IL-6, CXR4, and CXCL12 and the amplification of PIK3CA, Akt and bone morphogenetic protein (BMP) pathways have been associated with ovarian cancer EMT.^17,124,125^ The review by Tan *et al.* provides an in-depth look at epithelial ovarian cancer metastasis.^28^ Recent clinical studies and xenograft research have shown that side populations of ovarian cancer within the ascites display characteristics of CSCs.^126,127^ These ovarian CSCs have heightened chemoresistance, the ability to asymmetrically proliferate, and the capacity to self-renew.

Research done *in vivo* on the ascites of ovarian cancer patients has shown that the formation of non-adherent spheroids within the ascites may be correlated to the recurrence of the disease. These non-adherent spheroids express high levels of CSC markers EpCAM, STAT3, and Oct4, as well as CA125.^34^ The upregulation of ovarian stem cell markers CD44 and CD177/c-Kit has been shown to be attributed to side populations within the ascites.^128,129^ The ABC transporter protein ABCG2/BCRP1 has also been shown to have a high expression in ovarian cancer cells found within the ascites.^36,37,129^ From these investigations, it is evident that the ascites facilitate an enhanced expression of chemoresistance, stem cell-like behavior, and metastasis in ovarian cancer. Preliminary findings seem to suggest that mechanotransduction plays an important role in this shift of phenotype, as evident through the commonality of markers and pathways modulated both *in vitro* and *in vivo.* However, further proof is necessary to corroborate these findings and develop new targets for the next generation of ovarian cancer treatments. Future studies will integrate the *in vitro* and *in vivo* data to direct research into treatment regimens that take mechanotransduction into consideration.

## IV. CONCLUSION AND FUTURE DIRECTIONS

Over the last 20 years, a new narrative has begun to emerge implicating mechanotransduction in the metastasis of ovarian cancer and the promotion of a CSC-like side population within the ascites. A gap in our understanding of ovarian cancer pathology is evident; one that must be bridged before treatment of the disease can be improved. It is well known that isolation in the peritoneal cavity allows ovarian cancer to progress into more advanced stages of disease, as well as disseminate to distant parts of the body. Correspondingly, the peritoneal cavity is a dynamic space, one that continuously changes shape and stimulates ovarian cancer cells with high levels of shear stress. *In vitro* models that can simulate the microenvironment are necessary to explore the effects of mechanotransduction on ovarian cancer in detail. With *in vitro* mechanical stress bioreactors, stresses can be isolated, explored, and used as a platform to test drug efficacy.

When designing bioreactors for ovarian cancer mechanotransduction investigations, there are several additional factors that should be considered. Beyond force stimulation and application duration, the other cell types present in ascites may be an additional avenue of investigation. The cell type distribution within ascites typically consists of 37% lymphocytes, 29% mesothelial cells, 32% macrophages and <0.1% adenocarcinoma cells.^12^ Investigations using a coculture of ovarian cancer, stromal, and immune cell types should be performed concurrently with force stimulus found within the peritoneal cavity. With this combinatory approach, it will be possible to gain a more complete picture of the cancer microenvironment and ascertain potential avenues of treatment. Additionally, non-cell factors such as chemotaxis,^130^ 3D culture^94,131–136^, and hypoxia^137^ should be considered for future investigations, in conjunction with mechanical cues to create a microenvironment that can more fully recapitulate *in vivo* conditions. The study of ovarian cancer mechanotransduction promises to improve patient treatment through future investigations that utilize designs pertinent to the specific microenvironment.

The field of mechanotransduction in ovarian cancer is still growing. Future investigations are needed to accurately model the forces present in the peritoneal cavity. Computer aided simulations modeling shear stress in the ascites and direct measurements of tissue stiffness will provide a strong foundation for all future exploration into the mechanobiology of this field. Limited experiments have been performed to show how ECM stiffness may affect ovarian cancer, consequently, more robust studies are needed to show the role of stiffness in ovarian cancer biology. The few studies modeling shear stresses on ovarian cancer have shown promising results, where the promotion of EMT, chemoresistance and CSC surface markers is evident. These results have a wide impact on the future of ovarian oncology and the potential process for drug screening. Mechanotransduction might yet prove to be the key to improving the clinical outcomes in ovarian cancers.
